# Engineered mRNA-expressed bispecific antibody prevent intestinal cancer via lipid nanoparticle delivery

**DOI:** 10.1080/21655979.2021.2003666

**Published:** 2021-12-13

**Authors:** Lipei Wu, Weiwei Wang, Jiale Tian, Chunrun Qi, Zhengxin Cai, Wenhui Yan, Shihai Xuan, Anquan Shang

**Affiliations:** a Department of Laboratory Medicine, Dongtai People’s Hospital & Dongtai Hospital of Nantong University, Yancheng, P.R. China; b Department of Laboratory Medicine, Shanghai Tongji Hospital, School of Medicine, Tongji University, Shanghai, P.R. China; c Department of Pathology, Tinghu People’s Hospital, Yancheng, P.R. China; d Department of Laboratory Medicine, Tinghu People’s Hospital of Yancheng City, Yancheng, P.R. China

**Keywords:** PD-1, PD-L1, mRNA, LNP, bispecific antibody, cancer immunotherapy

## Abstract

The potential of antibodies, especially for the bispecific antibodies, are limited by high cost and complex technical process of development and manufacturing. A cost-effective and rapid platform for the endogenous antibodies expression via using the *in vitro* transcription (IVT) technique to produce nucleoside-modified mRNA and then encapsulated into lipid nanoparticle (LNP) may turn the body to a manufactory. Coinhibitory pathway of programmed death ligand 1 (PD-L1) and programmed cell death protein 1 receptor (PD-1) could suppress the T-cell mediated immunity. We hypothesized that the coblocking of PD-L1 and PD-1 via bispecific antibodies may achieve more potential antitumor efficacies compare with the monospecific ones. Here, we described the application of mRNA to encode a bispecific antibody with ablated Fc immune effector functions that targets both human PD-L1 and PD-1, termed XA-1, which was further assessed the *in vitro* functional activities and *in vivo* antitumor efficacies. The *in vitro* mRNA-encoded XA-1 held comparable abilities to fully block the PD-1/PD-L1 pathway as well as to enhance functional T cell activation compared to XA-1 protein from CHO cell source. Pharmacokinetic tests showed enhanced area under curve (AUC) of mRNA-encoded XA-1 compared with XA-1 at same dose. Chronic treatment of LNP-encapsulated XA-1 mRNA in the mouse tumor models which were reconstituted with human immune cells effectively induced promising antitumor efficacies compared to XA-1 protein. Current results collectively demonstrated that LNP-encapsulated mRNA represents the viable delivery platform for treating cancer and hold potential to be applied in the treatment of many diseases.

**Abbreviations:** IVT: *in vitro* transcription; LNP: lipid nanoparticle; hPD-1: human PD-1; hPD-L1: human PD-L1; ITS-G: Insulin-Transferrin-Selenium; Pen/Strep: penicillin-streptomycin; FBS: fetal bovine serum; TGI: tumor growth inhibition; IE1: cytomegalovirus immediate early 1; SP: signal peptide; hIgLC: human immunoglobulin kappa light chain; hIgHC: human IgG1 heavy chain; AUC: area under the curve; Cl: serum clearance; Vss: steady-state distributed volume; MLR: mixed lymphocyte reaction.

## 1. Introduction

Monoclonal as well as bispecific antibodies therapies have showed huge impact on treating cancer, autoimmune infectious and disorders [1,2]. However, the high cost and difficulty of large-scale production of antibodies, especially for bispecific antibodies, limits its application [3,4]. The preclinical development cycle of antibody drugs also takes at least 2–3 years [1]. Moreover, to maintain the plasma efficacious levels of some antibodies requires frequent injections due to the poor in vivo stability and rapid clearance by the proteases, and multiple injections may bring higher medical fee and more side effects [5,6]. In recent years, several studies reported that proteins, including antibodies, could be produced via the endogenous manner [6]. In brief, delivering *in vitro* transcribed mRNA may use the human body as a ‘manufacture factory’ for producing antibodies, which can simplify the complex processes and complete the posttranslational modification closer to human needs in somatic cells [5]. Moreover, unlike protein-based therapeutics, production of mRNA is simple and cost effective; high levels of therapeutic protein are produced, folded, and modified by host cells and the delivered mRNA is continuously translated for extended and controllable durations [7,8].

The keys to mRNA therapy are the mRNA molecule itself and the delivery system. mRNA molecule is easily degraded and holds immunogenicity and low translation efficiency [9,10]. Therefore, the *in vivo* safety and expression efficiency of mRNA could be mainly affected by sequence optimization and delivery system [10]. Lipid nanoparticles (LNPs) are the most clinically advanced nonviral gene delivery system [9]. LNPs could safely and effectively deliver nucleic acids, especially for mRNA, overcoming a major barrier preventing the development and use of genetic medicines [5,11]. In addition to negatively charged mRNA, LNP encapsulates four components: ionizable cationic phospholipids, neutral auxiliary phospholipids, cholesterol, and polyethylene glycol-modified phospholipids [12,13]. The effect of excipients in nanoparticles is similar to the effect of such excipients in liposomes: neutral auxiliary phospholipids are generally saturated phospholipids, which can increase the phase transition temperature of cationic liposomes, support the formation of lamellar lipid bilayers and stabilize their structural arrangement; cholesterol has strong membrane fusion and promotes mRNA intracellular intake and cytoplasmic entry; PEGylated phospholipids are located on the surface of nanoparticles, improve their hydrophilicity, avoid rapid clearance by the immune system, prevent particle aggregation, and increase stability [12]. To make IVT mRNA suitable for therapy, several qualities, including stability and translatability have been improved [14]. Several studies reported that optimized open reading frame (ORF), 3ʹ and 5ʹ UTR as well as long 3ʹ poly (A)-tail bring more stable IVT-mRNA and substantially higher in vivo expression level [15,16]. In addition, in order to make the mRNA increased translation without obvious non-immunogenicity, the purification and application of modified uridine [17]. Furthermore, lipid nanoparticles (LNPs) are currently most-efficient mRNA delivery system, which enables the high in vivo protein expression levels in liver tissues or other tumor tissues [9]. The rapid and accurate increase of drug concentration at the site where the antibody exerts its efficacy can theoretically achieve a lower dose and improve the efficacy compared with the traditional intravenous infusion of protein antibody [9]. It is very important that IVT-mRNA expression is completed in the cytoplasm without entering the nucleus, which minimizes the risk of inserting into the genome relative to DNA-based therapies [14].

PD-1, as one member of B7 family, is also an immune checkpoint, which is mainly expressed on the activated T-cells with ligands of PD-L1 and PD-L2 [18]. Upon ligation, a negative pathway is promoted to inhibit the function of activated T-cells via downregulating the signaling of TcR/CD28 [19]. The expression of PD-L1 on the surface of tumor cells has become a driver for tumor growth caused by tumor escape from immune cell pursuit [18]. Thus, the PD-1/PD-L1 pathway is seen as an important mechanistic axis adopted by tumors to facilitate tumor.

In this study, we proposed a hypothesis that bispecific antibody XA-1 would effectively suppress the progression of intestinal cancer. Here, the proof of concept was provided for XA-1 to cotarget PD-L1 and PD-1 and ablate Fc immune effector functions. XA-1 can simultaneously block both PD-1 and PD-L1 to result in complete blockade of PD-1/PD-L1 pathway and facilitate bridging between the PD-L1 positive tumor cells and PD-1-positive-activated T-cells in tumor microenvironment (TME). We applied the LNP based IVT-mRNA system for the delivery of XA-1, and demonstrated the bioactivities and functionalities via *in vitro* and *in vivo* assays.

## 2. Materials and methods

### 2.1. Reagents

CHO-K1, WIL2-S, AML-12, and MC38 cells were obtained from ATCC. CHO-K1 cells expressing either human PD-1 or PD-L1, aAPC/CHO-K1 cells (CS187110), and human PD-L1 positive aAPC/CHO-K1 cells (CS187108) were all brought from Shanghai Bangjing Industrial Co., Ltd (Shanghai, China). The XA-1 antibody gene sequences were cloned into the vector and expressed in CHO cells, then the proteins were purified via the protein A chromatography (Life Technologies). Human PD-1 (hPD-1) knock-in (C57BL/6 background) mice were brought from Shanghai Model Organisms Science and Technology Co., Ltd. (China, Shanghai). Human IL-2 and human IFN-γ ELISA kits were purchased from R&D System (USA). Recombinant PD-1, PDL1, and PD-L2 were obtained from R&D System (USA). Recombinant XA-1 purified from DNA plasmid or mRNA transfection in CHO or Expi293F cells, respectively, were performed according standard method for full length IgG antibody purification via protein A.

### 2.2. Cell culture

AML-12 cells were grown in a 1:1 mixture of DMEM and Ham’s F12 with Insulin-Transferrin-Selenium (ITS-G) medium supplement, 100 U/mL penicillin-streptomycin (Pen/Strep), and 10% fetal bovine serum (FBS) (Gibco). All cell lines were cultured at 37°C in 5% CO_2_. When the cells reached 70%–80% monolayer, they were detached from the flask using 0.25% Trypsin-EDTA solution and split 1:5.

### 2.3. In vitro *transcription and lipid-nanoparticle encapsulation of XA-1 mRNA*

The mRNAs encoding the heavy and light chain of XA-1 were prepared by applying the T7 RNA polymerase on the IVT templates (linearized plasmids), which was supplied by Genescript (Nanjing, China), with 5ʹ cap (Cap1) and a 3ʹ poly A tail containing more than 110 successive adenines. The 1-methylpseudourine-5′-triphosphate was applied to instead of UTP to obtain modified mRNA. Then the mRNAs with intact 5ʹ cap were purified by via MEGAClear RNA purification kits (Life Technologies, USA).

Both two mRNA were encapsulated in LNPs using a self-assembly process according to previously reported method. In brief, the aqueous solution of mRNA was adjusted to pH = 4.0 and then rapidly mixed with the lipids dissolved in ethanol. Then the LNPs contained the phosphatidylcholine, ionizable cationic lipid, PEG-lipid, and cholesterol at a ratio of 10:50:1.5:38.5 (mol/mol). Final concentration of LNP-mRNA was at of ~0.4 mg/mL and then stored at 4°C. The particle size of the mRNA-LNPs in this experiment was in the range of 70–90 nm and the entrapment efficiency was above 95%.

### 2.4. Affinity and binding specificity to PD-1/PD-L1

The binding affinities of purified XA-1 from the DNA plasmid in CHO cell or mRNA in HEK293T sources were both detected via surface plasmon resonance (SPR) method with BIAcore (Cytiva, USA). Above antibodies were captured onto a sensor chip CM5 at 5 μg/mL with the serially soluble human PD-1 or PD-L1 (2X from 25 nM to 0.39 nM) flowed in the running buffer. The HBS-EP+ buffer was used as running buffer, containing 0.01 M HEPES, 0.15 M NaCl, 3 mM EDTA, pH 7.4, while the regeneration buffer contained 10 mM glycine-HCl and the pH was 1.5 ~ 2.0. Further experimental details were performed according to the user’s manual. Equilibrium dissociation constant (KD) was calculated from the ratio of rate constants koff/kon.

### 2.5 In vitro *functional validation assays*

For the binding affinity of the CHO cells overexpressed the human PD-1 or PD-L1, the CHO cells were seeded into the U-bottom plates, then treated with two sources of XA-1 at final concentration between 0.003 tand 50 nM. XA-1 can bind to CHO overexpressed human PD-1 or PD-L1 cells were measured with PE labeled mouse anti-human Fc mAb (Acro biosystems) and the EC_50_ values were calculated via the GraphPad Prism 8.4.

For the flow cytometry analysis, test was performed in the CHO-K1 cells expressing either human PD-1 or PD-L1. These cells were stained in duplicate with Live Dead Zombie Green viability dye (BioLegend), then blocked with 1xDPBS containing 1%BSA after washing. Then incubated with AF647 conjugated ANTIBODIES for 60 min. The stained cell was further acquired on Cell Analyzer and the data was analyzed via FlowJo software. Flow cytometry gating and representative plots were then performed. The MFI value was plotted against the concentration of staining antibody and EC_50_ calculated using a log agonist vs. response-variable slope.

For antibody-dependent cellular cytotoxicity (ADCC) test, PD-1 or PD-L1 expressing CHO-K1 cells were plated in 96-well plate (5 × 10^3^ cells/well) in 100 μL of cell growth medium. After 15 h incubation at 37°C, 50 μL RPMI 1640 including 0.5%BSA was added. The antibodies from two sources were also added for 30-min incubation. Then incubated the FcγRIIIa positive Jurkat cells in target cells at 37°C for 6 h. The luminescence signals were detected via Promega Bio-Glo reagents. ADCC effector function assay with CD20-positive WIL2-S cells and the anti-CD20 antibodies as positive controls were also detected as described above.

For complement-dependent cytotoxicity (CDC) assay, the 5.0 × 10^4^ cells/well PD-L1/PD-1 CHO-K1 or CD20-positive WIL2-S cells were seeded in 96-well plate. All the antibodies were added in duplicate, and then incubated for 0.5 h. Human complements brought from Sigma were added for 60 min. The Invitrogen Alamar Blue reagent was also added then incubated for 24 h. Results were read at 560 nm and then the EC_50_ values were calculated via GraphPad Prism 8.4.

### 2.7. In vivo *expression validation in mice*

The LNP-XA-1 mRNA at the doses of 0.2, 0.6, and 2 mg/kg were intravenously (i.v.) treated to the female C57BL/6 or NOD/SCID mice weighing 18–20 g via tail veins. The mouse blood was collected from orbit or tail vein and then sit the samples at 4°C for 30 min, and furtherly centrifuged at 1000 g for 15 min. Serum was collected and applied for further ELISA detection or purification of antibodies.

### 2.8. In vivo *efficacy studies*

All hPD-1/hPD-L1 knock-in female mice were raised in the animal facility with the condition of pathogen-free. All animal research methods and studies were approved by the Institutional Animal Care and Use Committee and performed in SL biotechnology Co., Ltd. (Shanghai, China). Mice were housed in a 12 h dark/light cycle facility in cages (n = 6) with free to water and food. The 0.2 mL of MC38 cells (5*10^6^ cells/mL) were s.c. implanted into the right flank of mice. About 10 days postimplantation, the mice were randomly assigned with mean tumor volume ~ 100 mm^3^. On the day 11 and 23 postimplantation, these mice were i.v. injected with empty LNPs or mRNA-LNPs at the doses of 0.2, 0.6, and 2 mg/kg, using protein antibody at the dose of 10.0 mg/kg as positive control which was injected at day 11, 17, 23, and 29. The body weights and tumor volumes were measured twice a week. Tumor-bearing mice were euthanized when the body weight loss was over 20%. Detection of tumor size was performed via a standard digital caliper and the tumor growth inhibition (TGI) (%) was calculated as follow: 100% × (Tumor volume of mice treated with empty LNPs-Tumor volume of mice treated with agent)/(Tumor volume of mice treated with empty LNPs-Tumor volume of mice treated with empty LNPs before dosing). Mouse with < 14 mm^3^ tumor volume was considered as Complete Responders (CR). The % change in body weight (BW) was calculated by the formula (BW on observation day – BW on initial day)/BW initial day × 100%.

### 2.9. Statistical analysis

Statistical analysis was performed using GraphPad Prism software. Data are shown as the mean±SEM. One-way ANOVA followed by Tukey’s post hoc test was applied for multiple comparisons analysis. Two-way ANOVA followed by the Sidak multiple-comparisons test was applied for repeated measures analysis.

## 3. Results

In this study, we proposed a hypothesis that bispecific antibody XA-1 would effectively suppress the progression of intestinal cancer. Here, the proof of concept was provided for XA-1 to cotarget PD-L1 and PD-1 and ablate Fc immune effector functions. XA-1 can simultaneously block both PD-1 and PD-L1 to result in complete blockade of PD-1/PD-L1 pathway and facilitate bridging between the PD-L1 positive tumor cells and PD-1-positive-activated T-cells in tumor microenvironment (TME). We applied the LNP based IVT-mRNA system for the delivery of XA-1, and demonstrated the bioactivities and functionalities via *in vitro* and *in vivo* assays.

### 3.1. Design and optimization of XA-1 mRNA

**Figure 1.**
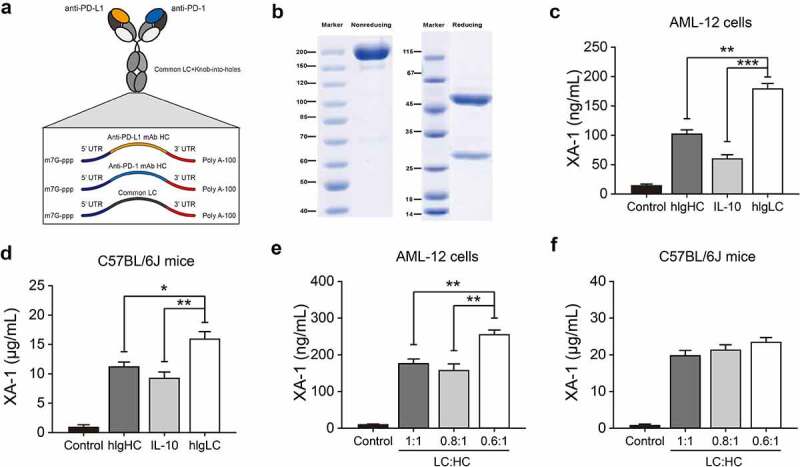
Design and optimization of XA-1 mRNA for further *in vivo* evaluation. (a) Schematic of the structure of mRNA-encoded the heavy and light chains of the XA-1. (b) SDS-PAGE detection of the XA-1 translated from mRNA in supernatants from Expi293F cells. The expression levels of XA-1 in (c) supernatants of AML-12 cell and (d) C57BL/6 J mice with different SPs. The expression levels of XA-1 in (e) AML-12 and (f) C57BL/6 J with different molar ratios. *p < 0.05, **p < 0.01, ***p < 0.001. Results were showed as SEM, n = 6

As the results showed in Figure 1a, the intact mRNA molecule is composed of a 5ʹ cap, 5ʹ and 3ʹ UTRs, an ORF, and a 3ʹ poly(A)-tail. In the present study, the cap of our designed mRNA was 2ʹ-O-methylated Cap0 (Cap1); the cytomegalovirus immediate early 1 (IE1) and human growth hormone gene were selected as 5ʹ and 3ʹ UTR, respectively. In addition, the length of 3ʹ poly (A)-tail was detected as 110 nt. All the optimization or selection of these elements are intended to promote the efficient expression of proteins *in vivo*. Moreover, signal peptide (SP), as a short polypeptide containing 5–30 amino acids, directs the translocation of newly synthesized secretory proteins to the secretory pathway. Based on the above selected mRNA elements, we optimized the SP in the protein coding sequence region via applying three SPs which originate from human immunoglobulin kappa light chain (hIgLC), interleukin-10 (IL-10), and human IgG1 heavy chain (hIgHC) for the *in vit*ro expression validation in HEK293T cells.

In current study, the integrity of XA-1 in supernatant of Expi293T cells transfected with mRNA-encoded the HC and LC of XA-1 with fixed SP was confirmed via SDS-PAGE analysis. As shown in Figure 1b, intact XA-1 as well as HC/LC chain were observed in nonreducing and reducing gel, respectively, with reasonable molecular weight. Then the mRNA encoded the HC and LC of XA-1 with three kinds of SPs were paired and transfected into AML-12 cells or delivery into C57BL/6 mice. The hIgLC SP resulted in higher expression of XA-1 in both *in vitro* AML-12 cells and *in vivo* C57BL/6 mice (Figure 1c-d). Therefore, hIgLC SP was selected and applied for subsequent optimization.

Different molar ratios of LC and HC were reported affected both *in vitro* and *in vitro* antibody expression. Moreover, our previous data indicated that translation efficiency of proteins decreases as the mRNA length increases (data not shown). Thus, we speculated the relatively slower heavy chain expression efficiency compared to that of light chain which may subsequently limit the assembly and secretion of IgG antibody. In current study, *in vitro* transfection of AML-12 cells with mRNA encoding the XA-1’s LC and HC at molar ratios of 1:1, 0.8:1, and 0.6:1, and the results demonstrated that the LC/HC molar ratio of 0.6:1 resulted in the highest antibody expression compared with the others (Figure 1e). However, *in vivo* results did not exhibit a significant higher ratio, although antibody expression level at the LC:HC ratio of 0.8:1 was slightly higher than 0.6:1 and similar to the 1:1 (Figure 1f). After comprehensive consideration, the mRNA of with hIgLC SP at the LC/HC molar ratio of 0.6:1 was chosen in the subsequent studies.

### 3.2. In vivo *expression and functionality of mRNA-encoded antibodies*

**Figure 2.**
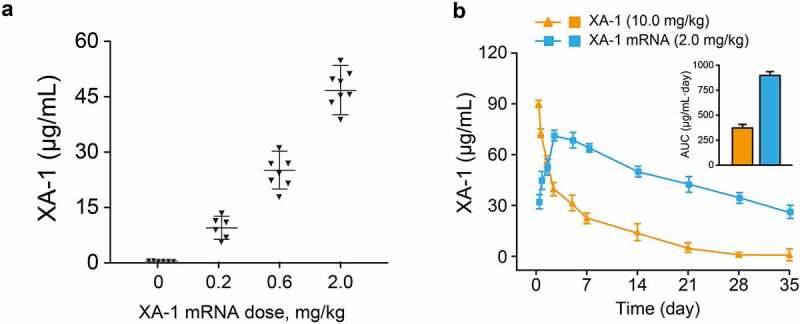
*In vivo* expression of XA-1 mRNA-LNPs in rodent animals. (a) Serum concentration of XA-1 in the C57BL/6 mice received single injection of XA-1 mRNA-LNPs at three three doses. (b) Pharmacokinetic comparison of XA-1 protein (10 mg/kg) and XA-1 mRNA-LNPs (2 mg/kg) in C57BL/6 mice. All the data were showed as Mean ± SEM, n = 6

**Figure 3.**
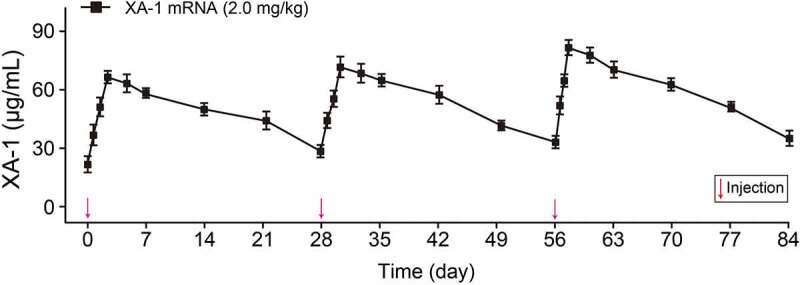
The serum concentrations of XA-1 in NOD/SCID mice received the three repeated injections of XA-1 mRNA-LNPs. All the data were showed as Mean ± SEM, n = 6

We further investigated whether the delivery of XA-1 mRNA is capable of expressing intact antibodies in rodent animals. Normal C57BL/6 mice received the single injection of formulated XA-1 mRNA at the doses of 0.2, 0.6, and 2 mg/kg did not observed obvious side effects. As the results shown in Figure 2a, the serum levels of XA-1 at all three doses clearly increased after single dose administration. Single injection of 10 mg/kg XA-1 protein and 2 mg/kg LNP-XA-1 mRNA in C57BL/6 J mice were performed to compare the pharmacokinetic parameters of endogenously translated XA-1 with the XA-1 from the CHO cell source. As shown in Figure 2b, serum concentration of XA-1 protein rapidly peaked after injection, then dramatically decreased within two days, and continued to slowly decrease over next three weeks. In contrast, the serum levels of XA-1 that was endogenously translated from mRNA peaked at 48 h (~75 μg/mL) and remained over 30 μg/mL at least four weeks. Moreover, the XA-1 endogenously translated from single dose of mRNA-LNPs also exhibited a higher value of area under the curve than that of XA-1 protein. Not only that, the sustained serum concentrations of XA-1 from the DIW injection of 2.0 mg/kg LNP-mRNAs were exhibited in almost consistent concentration-time curves, indicating that the successive injection of XA-1 mRNA-LNPs in mice is capable of continuously expressing complicated bispecific antibodies (Figure 3).

### 3.3. Functional activity of mRNA-encoded XA-1 antibodies

**Figure 4.**
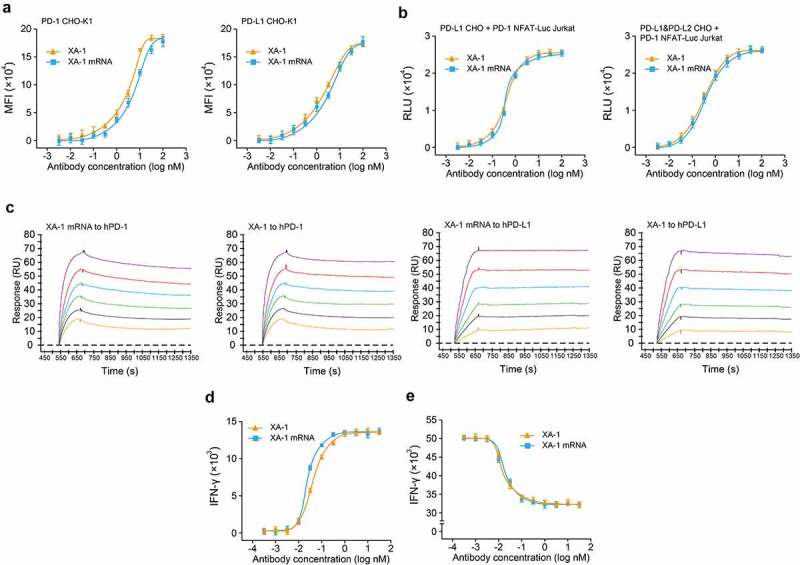
*In vitro* functional activity of mRNA translated XA-1. (a) Binding of XA-1 from two sources to CHO-K1 cells expressing cell surface PD-1 or PD-L1 by flow cytometry. (b) NFAT reporter assay. (c) Association and dissociation of Pembrolizumab to hPD-1 and hPD-L1 by SPR. (d) IFN-γ levels. (e) Tumor cell line killing tests. All the data were showed as Mean ± SEM, n = 6 for Figure (a, b, d, e)

The *in vitro* functional activities of exogenously translated XA-1 from mRNA to bind or block the PD-1 pathway, as well as enhance T cells activities, were evaluated by cell-based assays compared to the XA-1 proteins from CHO cells. Specific binding of XA-1 to PD-1 or PD-L1 expressing cells were evaluated using flow cytometry and the CHO cells engineered to express each targeted antigen. Current results showed in Figure 4a demonstrated that the specific binding of XA-1 to each target, and the binding affinity of XA-1 from mRNA source for each target was largely preserved relative to XA-1 protein from CHO cell source (PD-1 EC_50_: 0.86 nM for mRNA source, 0.48 nM for CHO source; PD-L1 EC_50_: 0.58 nM for mRNA source, 0.38 nM for CHO source). The Fc effector function of XA-1 was evaluated in the PD-1 and PD-L1 expressing CHO cells, and no ADCC or CDC (Table 1) activities were observed. Moreover, no specific binding of XA-1 to human Fcγ receptor (FcγR) was observed in ELISA, which was similar to that of the XA-1 protein form CHO source (Table 2).

**Table 1.** Human ADCC and CDC activity EC_50_ (nM) of XA-1 from mRNA-translated sources

Antibody	WIL2-S	PD-L1 CHO-K1	PD-1 CHO-K1	CHO-K1
ADCC				
XA-1 mRNA	NA	>1000	>1000	>1000
XA-1	NA	0.015	>1000	>1000
Rituximab	0.039	NA	NA	NA
CDC				
XA-1 mRNA	NA	>1000	>1000	>1000
XA-1	NA	>1000	>1000	>1000
Rituximab	2.12	NA	NA	NA

**Table 2.** Human Fcγ receptor and C1q binding EC_50_ (nM)

Antibody	FcγRIa	FcγRIIa(H)	FcγRIIb	FcγRIIIa(V)	C1q
XA-1 mRNA	>1000	>1000	>1000	>1000	>1000
XA-1	>1000	>1000	>1000	>1000	>1000
Rituximab	1.09	20.11	84.3	19.43	26.11

Moreover, SPR detection exhibited similar binding affinity of the XA-1 from mRNA or CHO cell source with the KD of 0.19 nM and 0.38 nM for hPD-1, 0.08 nM and 0.12 nM for hPD-L1, respectively (Figure 4b). The NFAT-driven luciferase reporter assay and primary T cell assay were performed to assess the abilities of mRNA-translated XA-1 to functionally block the PD-1/PDL1 pathway. For the NFAT-driven luciferase reporter assay, Jurkat cells, which stably express hPD-1 and NFAT reporter were cocultured with PD-L1 or dual PD-L1 + PD-L2 stable expressing CHO cells in the presence of two sources of XA-1. NFAT reporter expression increased significantly in response to both two sources of XA-1 treatment in both PD-L1 (*P* > 0.05) and PD-L1 + PD-L2 (*P* > 0.05) expressing cells without significant difference (Figure 4c).

The functional activity of XA-1 from two sources was further evaluated in a mixed lymphocyte reaction (MLR). In this assay, the allogeneic response of primary T cells against allogeneic DCs can be suppressed by blocking the PD-1 pathway. As assessed by both cytokine and cytotoxicity measurements, both two kinds of XA-1 treatment were effective in PD-1 pathway inhibition with comparable EC_50_ values of IFN-γ stimulation in Figure 4d (EC_50_ = 0.06 nM for mRNA source, EC_50_ = 0.08 nM for CHO source) and tumor cell line killing in Figure 4e (EC_50_ = 0.038 nM for mRNA source, EC_50_ = 0.022 nM for CHO source).

### 3.4. Antitumor efficacy of XA-1 mRNA-LNPs in humanized mouse tumor models

In order to confirm whether the *in vivo* mRNA-translated XA-1can retain the anti-cancer efficacies of XA-1 protein from CHO cell sources, we further used the humanized tumor xenograft mouse models to investigate its inhibitory and protective effects on tumor growth and animal survival rate, respectively.

**Figure 5.**
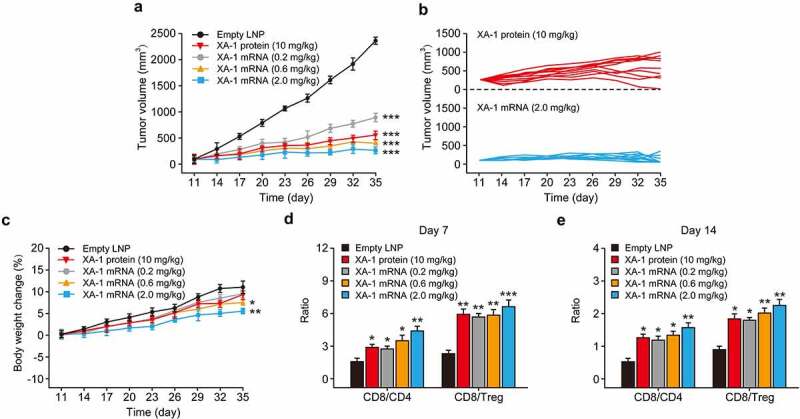
*In vivo* antitumor efficacy test of LNP-Pembrolizumab mRNA in hPD-1 and hPD-L1 knock-in mouse model. (a–b) Tumor growth inhibition (TGI), (c) body weight changes and (d–e) changes in ratios of tumor infiltrating CD4^+^ T cells, CD8^+^ T cells, and Treg cells of tumor bearing mice treated with XA-1 mRNA-LNPs at the doses of 0.2, 0.6, and 2.0 mg/kg using XA-1 proteins and empty LNPs as control. Red and blue arrows indicated the injection day of XA-1 mRNA-LNPs or XA-1 proteins, respectively. All the data were showed as Mean ± SEM, n = 10

In the human PD-1 and PD-L1 knock in MC38 tumor model, weekly treatment of XA-1 mRNA-LNPs at the doses of 0.2, 0.6, and 2.0 mg/kg resulted in significantly enhanced tumor growth inhibition (TGI), while empty LNPs treatment exhibited no significant antitumor effect (Figure 5a). Moreover, XA-1 mRNA-LNPs at 0.2, 0.6, and 2.0 mg/kg exhibited the TGI of 69%, 85%, and 94.1%, respectively, while XA-1 protein group at the doses of 10 mg/kg was 80.2%, compare to the empty LNPs and these inhibitions were all statistically significant (all *P* < 0.001). In addition, significantly outstanding antitumor efficacy was appeared in the model group received the XA-1 mRNA-LNPs at the dose of 2.0 mg/kg group with 7/10 mice achieved tumor free, while this of XA-1 protein group was only 1/10 (Figure 5b). Furthermore, only slight body weight loss after each single injection were observed, and then rapidly recovery within two days (Figure 5c).

Further to assess the therapeutic immune regulation, the ratios of CD4^+^ and CD8^+^ T cells at 7 and 14 days after first treatment of XA-1 mRNA-LNPs were assessed. As the results showed in Figure 5d-e, flow cytometry analysis exhibited increased the frequency of CD4^+^ and CD8^+^ T cells, whereas the ratios of CD8/Treg and CD8/CD4 cells were significantly increased in groups received the treatment of all three doses of XA-1 mRNA-LNPs compared to the empty LNPs.

## 4. Discussion

In recent years, mRNA-based therapeutic agents have been applied in clinical trials in different therapy ideas, including infectious disease vaccines, cancer vaccines as well as intratumoral injection therapy [20,21]. As a direction for future development, mRNA-based protein replacement therapy has also received a lot of attention [20]. In brief, recent research demonstrated that the intravenous delivery of mRNA could realize the rapid and effective expression of proteins as well as antibodies, and further overcome the challenges of chemical manufacturing and control [22,23]. The expression of antibodies in the form of mRNA can improve the kinetic properties of some antibodies, but also target intracellular targets [7]. In the treatment of liver-related diseases, the therapeutic window can also be improved by reducing the chance of systemic exposure through in situ synthesis and secretion by the liver [24,25]. Specifically, application of LNP-formulated mRNAs also could effectively address the disadvantages of bispecific antibodies, including poor *in vivo* stability and CMC challenges [22]. Moreover, endogenous production of mRNA-translated antibodies makes low cost and simple manufacturing process compared to antibodies produced via host translational machinery [26]. More importantly, the application of LNP-mRNAs can enable local expression of antibodies or proteins without the need of structure optimization process [27,28]. Previously reported data demonstrated the successful application of IVT-mRNA for endogenous expression of antibodies and exhibited prominent advantages in protecting humanized mice from HIV-1 challenge and selectively reduced the volume of HER2-positive tumors [5,29].

In present research, we described the application of IVT-mRNA to encode a bispecific antibody which targets both human PD-L1 and PD-1 to completely block inhibitory interactions between PD-1 pathway molecules for treating solid tumors, and termed XA-1. In current study, we optimized the signal peptide and LC/HC molar ratio of XA-1 mRNA, and further investigated whether the delivery of optimized XA-1 mRNA-LNPs could endogenously express XA-1 antibodies in normal C57BL/6 mice. Firstly, these mice received the single intravenous injection of formulated XA-1 mRNA at the doses of 0.2, 0.6, and 2 mg/kg were not observed with obvious side effects during the entire experimental period. Clearly dose-dependent upregulation of the serum levels of XA-1 were observed at 24 h after three doses of mRNA-LNP administration. Moreover, normal C57BL/6 mice received the single injection of XA-1 mRNA-LNPs at the dose of 2 mg/kg produced comparable serum XA-1 level with those ones received the 10 mg/kg of XA-1 proteins. More importantly, significantly prolonged XA-1 persistence in the blood circulation were observed after the single intravenous injection of XA-1 mRNA-LNPs compared to the 10 mg/kg of XA-1 antibodies from CHO sources.

The duration at which serum levels of antibodies remain above therapeutic concentrations *in vivo* determines the antitumor effect in some cases. After single treatment, serum levels of endogenously translated XA-1 at 24 h was comparable with that of XA-1 antibodies. Then, the serum XA-1 levels in those mice received XA-1 antibodies continuously declined to baseline within three weeks, while that of mice administrated with XA-1 mRNA-LNPs was maintained rising until 72 h with the C_max_ of ~75 μg/mL, and further remained over 35 μg/mL for more than 21 days. Our current data demonstrated that single injection of XA-1 mRNA-LNPs resulted in more robust serum antibody levels with enhanced duration compared the XA-1 antibodies from CHO sources. It should be noticed that the serum levels of endogenously translated XA-1 tend to be sustained longer than the purified agents from CHO cells, which is mainly due to the differences in the delivery method, translated efficiency as well as endogenous modification. The difference between antibodies produced by endogenous hepatocytes and exogenous CHO cells is exactly the problem that needs to be explored in subsequent studies. It is worth mentioning that we did not observe antidrug antibody (ADA) response, which is possible that ADA production by single injection of humanized antibodies occurs in mice. While the immunodeficient mice were used in the repeated injection test because of the potential immunogenicity of the humanized antibody under three repeated injections. Above results demonstrated that treatment of mRNA encoding therapeutic proteins or antibodies can result in the serum antibody levels not less than corresponding purified agents. In short, serum levels of endogenously expressed antibodies tend to be sustained longer than the purified agents, which is mainly due to the differences in the delivery method, translated efficiency as well as endogenous modification. The difference between antibodies produced by endogenous hepatocytes and exogenous CHO cells is exactly the problem that needs to be explored in subsequent studies. We also plan to purify the mRNA-encoded antibodies expressed in mice in subsequent experiments and compare them with antibodies expressed in CHO cells.

Further a series of *in vitro* and *in vivo* experiments were performed to confirm whether the endogenously mRNA-translated XA-1 maintained the comparable *in vitro* functionalities compared with XA-1 from CHO cell source, and whether also could effectively inhibit the tumor growth of tumor-bearing mice. XA-1 may exhibit more comprehensive blockade of PD-1/PDL2 pathway via bridging T cells (PD-1) and tumor cells (PD-L1) which also results in enhanced effector T cell activation and tumor cell killing. Our results showed that each arm of XA-1 from both mRNA and CHO cell sources retained comparable and robust binding affinities for PD-1 or PD-L1, and could fully block the binding of PD-1 to both PD-L1 and PD-L2. The mRNA-translated XA-1 resulted in enhanced *in vitro* activation of T cells at comparable concentration relative to the XA-1 antibodies, which enabled the bridges of the cells stably expressing PD-1 and PD-L1. Above results collectively indicated that the XA-1 from mRNA-translated source hold similar functional *in vitro* pharmacological characters to the XA-1 antibodies purified from CHO cells.

XA-1 mRNA-LNPs therapy also resulted in promising antitumor efficacies in humanized tumor xenograft models reconstituted with human T cells or PBMCs. Single i.v. injection of 0.2, 0.6, and 2.0 mg/kg of XA-1 mRNA-LNPs for 6 weeks obviously inhibited the growth of colorectal carcinoma and significantly improved the survival rate of tumor bearing mice. Not only that, the prolonged persistence of endogenously translated XA-1 in serum holds potential to decrease the clinical dose, injection frequency as well as the treatment costs.

## 5. Conclusion

In summary, our study demonstrated that the treatment of XA-1 mRNA-LNPs could effectively express endogenous therapeutic bispecific antibodies via hepatocytes providing an alternative to direct antibody treatment for treating cancer. Not only that, current strategy of protein replacement could also be applied for other targets.

## Highlight

- A mRNA-encoded bispecific antibody (XA-1) targeted PD-1 and PD-L1 has been created.
- A single dose of XA-1 mRNA-LNPs exerted notably prolonged XA-1 persistence in vivo.
- XA-1 mRNA-LNPs therapy obviously inhibited the growth of colorectal carcinoma.

## References

[1] Leavy O. Therapeutic antibodies: past, present and future. Nat Rev Immunol. 2010;10(5):297. 20422787 10.1038/nri2763

[2] Tilg H, Jalan R, Kaser A, et al. Anti-tumor necrosis factor-alpha monoclonal antibody therapy in severe alcoholic hepatitis. J Hepatol. 2003;38:419–425. 12663232 10.1016/s0168-8278(02)00442-7

[3] Parren PWHI, Lugovskoy AA. Therapeutic antibody engineering: current and future advances driving the strongest growth area in the pharmaceutical industry. MAbs. 2013;5:175–177.

[4] Stadler CR, Bähr-Mahmud H, Celik L, et al. Elimination of large tumors in mice by mRNA-encoded bispecific antibodies. Nat Med. 2017 Jul;23(7):815–817. 28604701 10.1038/nm.4356

[5] Pardi N, Secreto AJ, Shan X, et al. Administration of nucleoside-modified mRNA encoding broadly neutralizing antibody protects humanized mice from HIV-1 challenge. Nat Commun. 2017;8:14630. 28251988 10.1038/ncomms14630 PMC5337964

[6] Kose N, Fox JM, Sapparapu G, et al. A lipid-encapsulated mRNA encoding a potently neutralizing human monoclonal antibody protects against chikungunya infection. Sci Immunol. 2019;4:eaaw6647. 31101672 10.1126/sciimmunol.aaw6647 PMC6629435

[7] Van Hoecke L, Roose K. How mRNA therapeutics are entering the monoclonal antibody field. J Transl Med. 2019;17:54. 30795778 10.1186/s12967-019-1804-8 PMC6387507

[8] Arya S, Lin Q, Zhou N, et al. Strong immune responses induced by direct local injections of modified mRNA-Lipid nanocomplexes. Mol Ther Nucleic Acids. 2020;19:1098–1109. 32059336 10.1016/j.omtn.2019.12.044 PMC7016160

[9] Ickenstein LM, Garidel P. Lipid-based nanoparticle formulations for small molecules and RNA drugs. Expert Opin Drug Deliv. 2019;16:1205–1226. 31530041 10.1080/17425247.2019.1669558

[10] Yu A M, Choi YH, Tu M J. RNA drugs and RNA targets for small molecules: principles, progress, and challenges. Pharmacol Rev. 2020;72(4):862–898. 32929000 10.1124/pr.120.019554 PMC7495341

[11] Rybakova Y, Kowalski PS, Huang Y, et al. mRNA delivery for therapeutic Anti-HER2 antibody expression In Vivo. Mol Ther. 2019;27(8):1415–1423. 31160223 10.1016/j.ymthe.2019.05.012 PMC6698250

[12] Guevara ML, Persano F, Persano S. Advances in lipid nanoparticles for mRNA-Based cancer immunotherapy. Front Chem. 2020;8:589959. 33195094 10.3389/fchem.2020.589959 PMC7645050

[13] Samaridou E, Heyes J, Lutwyche P. Lipid nanoparticles for nucleic acid delivery: current perspectives. Adv Drug Deliv Rev. 2020;154-155:37–63. 32526452 10.1016/j.addr.2020.06.002

[14] Miliotou AN, Papadopoulou LC. In Vitro-Transcribed (IVT)-mRNA CAR therapy development. Methods Mol Biol. 2020;2086:87–117. 31707670 10.1007/978-1-0716-0146-4_7

[15] Pardi N, Tuyishime S, Muramatsu H, et al. Expression kinetics of nucleoside-modified mRNA delivered in lipid nanoparticles to mice by various routes. J Control Release. 2015;217:345–351. 26264835 10.1016/j.jconrel.2015.08.007 PMC4624045

[16] Kamura T, Katsuda Y, Kitamura Y, et al. G-quadruplexes in mRNA: a key structure for biological function. Biochem Biophys Res Commun. 2020;526:261–266. 32209257 10.1016/j.bbrc.2020.02.168

[17] Rauch S, Lutz J, Kowalczyk A, et al. RNActive® technology: generation and testing of stable and immunogenic mRNA vaccines. Methods Mol Biol. 2017;1499:89–107. 27987144 10.1007/978-1-4939-6481-9_5

[18] Jiang X, Wang J, Deng X, et al. Role of the tumor microenvironment in PD-L1/PD-1-mediated tumor immune escape. Mol Cancer. 2019;18:10. 30646912 10.1186/s12943-018-0928-4 PMC6332843

[19] Sun L, Li CW, Chung EM, et al. Targeting glycosylated PD-1 induces potent antitumor immunity. Cancer Res. 2020;80:2298–2310. 32156778 10.1158/0008-5472.CAN-19-3133 PMC7272274

[20] Schlake T, Thran M, Fiedler K, et al. mRNA: a novel avenue to antibody therapy? Mol Ther. 2019 Apr 10;27(4):773–784. 30885573 10.1016/j.ymthe.2019.03.002 PMC6453519

[21] Reichmuth AM, Oberli MA, Jaklenec A, et al. mRNA vaccine delivery using lipid nanoparticles. Ther Deliv. 2016;7:319–334. 27075952 10.4155/tde-2016-0006 PMC5439223

[22] Stadler CR, Bähr-Mahmud H, Celik L, et al. Elimination of large tumors in mice by mRNA-encoded bispecific antibodies. Nat Med. 2017;23:815–817. 28604701 10.1038/nm.4356

[23] Sahin U, Karikó K, Türeci Ö. mRNA-based therapeutics–developing a new class of drugs. Nat Rev Drug Discov. 2014;13:759–780. 25233993 10.1038/nrd4278

[24] Li J, Gong YM, Wu J, et al. Anti-tumor necrosis factor-α monoclonal antibody alleviates parenteral nutrition-associated liver disease in mice. JPEN J Parenter Enteral Nutr. 2012;36:219–225. 22275328 10.1177/0148607111424412

[25] Trepotec Z, Lichtenegger E, Plank C, et al. Delivery of mRNA therapeutics for the treatment of hepatic diseases. Mol Ther. 2019;27:794–802. 30655211 10.1016/j.ymthe.2018.12.012 PMC6453508

[26] Huang H, Zhang C, Yang S, et al. The investigation of mRNA vaccines formulated in liposomes administrated in multiple routes against SARS-CoV-2. J Control Release. 2021;335:449–456. 34029632 10.1016/j.jconrel.2021.05.024 PMC8139338

[27] Tiwari PM, Vanover D, Lindsay KE, et al. Engineered mRNA-expressed antibodies prevent respiratory syncytial virus infection. Nat Commun. 2018;9:3999. 30275522 10.1038/s41467-018-06508-3 PMC6167369

[28] Kowalski PS, Rudra A, Miao L, et al. Delivering the messenger: advances in technologies for therapeutic mRNA delivery. Mol Ther. 2019;27:710–728. 30846391 10.1016/j.ymthe.2019.02.012 PMC6453548

[29] Hu W, Kaminski R, Yang F, et al. RNA-directed gene editing specifically eradicates latent and prevents new HIV-1 infection. Proc Natl Acad Sci USA. 2014;111:11461–11466. 25049410 10.1073/pnas.1405186111 PMC4128125

